# Editorial: Learning politics in a digitized world: when do social media foster political literacy?

**DOI:** 10.3389/fpsyg.2024.1433144

**Published:** 2024-06-20

**Authors:** German Neubaum, Anne Oeldorf-Hirsch, Teresa K. Naab

**Affiliations:** ^1^Department of Human-Centered Computing and Cognitive Science, University of Duisburg-Essen, Duisburg, Germany; ^2^Department of Communication, University of Connecticut, Storrs, CT, United States; ^3^Institute for Media and Communication Studies, University of Mannheim, Mannheim, Germany

**Keywords:** political learning, social media use, political knowledge, civic education and digital activism, political participation

The impact of social media technologies on their users' political learning and participation has been examined and documented extensively, especially in the last decade. Two questions have been guiding research in this field. First, do users gain political knowledge when they are exposed to platforms such as Instagram, TikTok, X, or Facebook? Second, does the use of social media encourage individuals to participate in the political realm? While most research revealed that exposure to those technologies goes hand in hand with people's participation in civic and political life, the link between use and enhanced political knowledge has yielded mixed findings, leading to the idea that there might not exist a practically relevant effect.

Implicitly, the focus on those two questions pursues the premise that if individuals can gain new knowledge through those emerging technologies, they will be able to engage in political processes. This Research Topic integrates scholarship to move this field forward by grasping the largely neglected psychological processes that connect media use, knowledge, and political action. The study of these psychological processes requires scholars to systematically account for characteristics of media content, sources, networks, the filtering effect of algorithms, users' motivation, skills, pre-existing views, and diverse forms of social media use. With this Research Topic, we offer a series of studies pointing to various relevant factors in those intermediated processes.

## Diving deeper into social media use–political knowledge link

Hopp and Kazmi clarify the connection between social media use and political knowledge by indicating that this relationship could be conditional. A key variable, according to them, may be misinformation and disinformation self-efficacy beliefs. Their three-study examination surprisingly uncovered that those with stronger self-efficacy beliefs and more frequent social media use have less political knowledge. They explain this effect by the concept of overconfidence, suggesting that higher self-efficacy leads people to process and internalize less political information because they feel they already know enough.

Schäfer and Schemer reveal that compared to consuming TV news or quality press newspapers, which is associated with more objective political knowledge, using social media news is not. Instead, the latter is linked to enhanced levels of *perceived* knowledge. Thus, using these platforms may mislead users to believe that they are informed while they are not. What is striking is that perceived knowledge is more strongly associated with online and offline participation than actual knowledge.

Dreston and Neubaum investigate that same triangle between social media news use, knowledge, and participation. In addition to replicating results from Schäfer and Schemer (indicating that social media news use is more strongly associated with subjective than with objective knowledge), they provide a nuanced view about which forms of social media use are connected with different dimensions of knowledge. They show that intentional news use is especially related to subjective knowledge, provided that users also engage in discussions on those topics.

## Pointing to the intervening power of media literacy in political learning effects

Citizens can only be well-informed if they are able to process accurate information from reliable and integer sources. They have to evaluate information and its sources accurately, that is, “source recognition” is key here. To be suspicious of sources that are not familiar can be an approach to dealing with the multitude of sources and content within those platforms. Sude et al.  examine circumstances under which individuals are able and motivated to identify falsehoods and questionable sources. Based on two consecutive experiments, they show that news media literacy helps users evaluate the accuracy of the content and their familiarity with the news outlet. Still, those with stronger partisan commitments appear to be more susceptible to (alternative) sources that promote partisan-favoring news. Thus, overcoming the temptation to fall for disinformation that favors one's pre-existing views represents a highly pivotal skill when using social media in the context of political information.

## Balancing the opportunities and risks of social media as facilitators of civic education

Schmitt et al. systematically analyze the chances and challenges that social media technologies provide as informal facilitators of civic education in terms of equipping citizens with knowledge, skills, abilities, and willingness to participate in political processes. They outline that new forms of self-directed learning through those technologies come with a loss of control over (quality and accuracy of) content and sources as well as dissemination procedures (e.g., filtering algorithms fostering social learning biases). In spaces in which everyone can be a provider of civic education, they propose that we need comprehensive frameworks with clearly defined criteria to evaluate the content and sources within social media.

This Research Topic of articles corroborates the informative value of systematically asking who (sources, recipients, and networks), what (specifics of the content and the context of the content), when (intervening factors such as motivation and skills), and how (different types of use) when it comes to studying political learning effects of social media use. Such a systematic approach will help this inevitably interdisciplinary line of inquiry to move our scholarly interest from asking, “do social media increase political knowledge and participation?” to “under which conditions can social media users gain new political knowledge, develop new civically and politically relevant skills, as well as increase their efficacy and motivation to participate politically?” Comprehensive investigations of this question also require further accounting for the (a) interconnectedness and relationships between actors (e.g., interpersonal relationships between users who share political information with one another and their mutual trust), (b) dynamic interaction between humans and technology (e.g., self-reinforcing processes between users' preferences and curating algorithms), and (c) “black box” between knowledge and action (considering that individuals need skills to, e.g., process, internalize, and share information in face of an abundance of social media affordances). This multi-layered approach, in our view, represents a promising step forward in disentangling the contingent effects those technologies can have on our political life.

## Author contributions

GN: Writing – original draft, Writing – review & editing. AO-H: Writing – original draft, Writing – review & editing. TN: Writing – original draft, Writing – review & editing.

